# A vaccine against cytomegalovirus: how close are we?

**DOI:** 10.1172/JCI182317

**Published:** 2025-01-02

**Authors:** Sallie R. Permar, Mark R. Schleiss, Stanley A. Plotkin

**Affiliations:** 1 Department of Pediatrics, Weill Cornell Medical Center, New York, New York, USA.; 2 Division of Pediatric Infectious Diseases and Immunology, University of Minnesota, Minneapolis, Minnesota, USA.; 3 Department of Pediatrics, University of Pennsylvania, Philadelphia, Pennsylvania, USA.; 4 Vaxconsult, Doylestown, Pennsylvania, USA.

## Abstract

The pursuit of a vaccine against the human cytomegalovirus (HCMV) has been ongoing for more than 50 years. HCMV is the leading infectious cause of birth defects, including damage to the brain, and is a common cause of complications in organ transplantation. The complex biology of HCMV has made vaccine development difficult, but a recent meeting sponsored by the National Institute of Allergy and Infectious Diseases in September of 2023 brought together experts from academia, industry, and federal agencies to discuss progress in the field. The meeting reviewed the status of candidate HCMV vaccines under study and the challenges in clinical trial design in demonstrating efficacy against congenital CMV infection or the reduction of HCMV disease following solid organ transplantation or hematopoietic stem cell transplantation. Discussion in the meeting revealed that, with the numerous candidate vaccines that are under study, it is clear that a safe and effective HCMV vaccine is within reach. Meeting attendees achieved a consensus opinion that even a partially effective vaccine would have a major effect on the global health consequences of HCMV infection.

## Introduction

For over 50 years, efforts have been made to develop a vaccine to prevent a viral infection that causes thousands of congenital abnormalities throughout the world each year and is also a common cause of infectious complications for patients receiving solid organ transplantation (SOT) and/or hematopoietic stem cell transplantation (HSCT). Human CMV (HCMV) is the herpesvirus responsible for these problems. The fact that HCMV is a frequent cause of both intrauterine infection and infectious complications of SOT has been well known for years, but aside from the use of antivirals, little could be done to prevent the complications of infection. At a recent international meeting sponsored by the National Institute of Allergy and Infectious Diseases (NIAID) on the status of vaccine development against HCMV scientists in the field came to an optimistic consensus that a preventative HCMV vaccine is feasible and that the clinical trials of multiple candidates in development should proceed, and indeed should accelerate, toward the goal of licensure.

HCMV vaccine development is complicated by the fact that immunity to infection is, even under the best of circumstances, incomplete. This fact is a major obstacle to the development of a vaccine to protect against congenital CMV (cCMV), since preconception immunity is not sufficient to protect the pregnant person against reinfection, with subsequent vertical transmission. However, in the context of pregnancy, there are data than clearly demonstrate a reduced risk of transmission in recurrent infection compared with primary maternal infection. HCMV transmission occurs in approximately 30%–40% of pregnancies affected by primary infection (1, 2), compared with 1.4% of pregnancies with recurrent infection (3). In a cohort study of approximately 3,500 multiparous women from a population with a high rate of cCMV infection, preconception seropositive status was associated with a 69% reduction of the risk of cCMV in future pregnancies (4). Other studies have indicated a protective effect of maternal immunity against cCMV infection, with significantly reduced rates of vertical transmission noted in women with nonprimary compared with primary infections (5, 6). Thus, although it is imperfect, preconception maternal immunity to HCMV is a barrier to vertical transmission. In addition to reducing the risk of transmission, there is some evidence demonstrating that HCMV-related sequelae are reduced, even if vertical transmission occurs, in infants born to women who are seropositive prior to pregnancy (7), compared with women with primary infection in pregnancy. Demonstrating whether an HCMV vaccine will recapitulate this partially protective effect on long-term neurodevelopmental outcomes will not be revealed in any phase III efficacy study that focuses on virus acquisition as an endpoint (discussed later in this Review), but such a benefit may stand as the most meaningful result from licensure of a vaccine against cCMV.

With respect to transplantation, the focus is to develop a vaccine strategy that could have several goals. Although the availability of effective prophylactic and preemptive antiviral therapy has made HCMV a rare cause of mortality in the transplantation setting, both HCMV-seropositive transplant recipients and HCMV-seronegative recipients of a positive graft demonstrate increased mortality when compared with HCMV-seronegative recipients with a HCMV-seronegative donor (8). HCMV seropositivity is also an important risk factor for impaired graft survival, increased risk of graft-versus-host disease, and other opportunistic infections such as invasive fungal infections. Antivirals are dependent on adherence and may be associated with significant toxicities, and antiviral resistance is a major concern in patients on long-term therapy (9). Thus, vaccination strategies may be of value in further decreasing the incidence and severity of HCMV disease as well as these other complications of transplantation. Such vaccines could be administered to either the transplant recipient or to the HSCT or living-related solid organ donor prior to transplantation. Whether the same vaccine(s) that might prove successful in this patient population would protect against congenital HCMV transmission in women of child-bearing age is uncertain.

**Figure 1:**
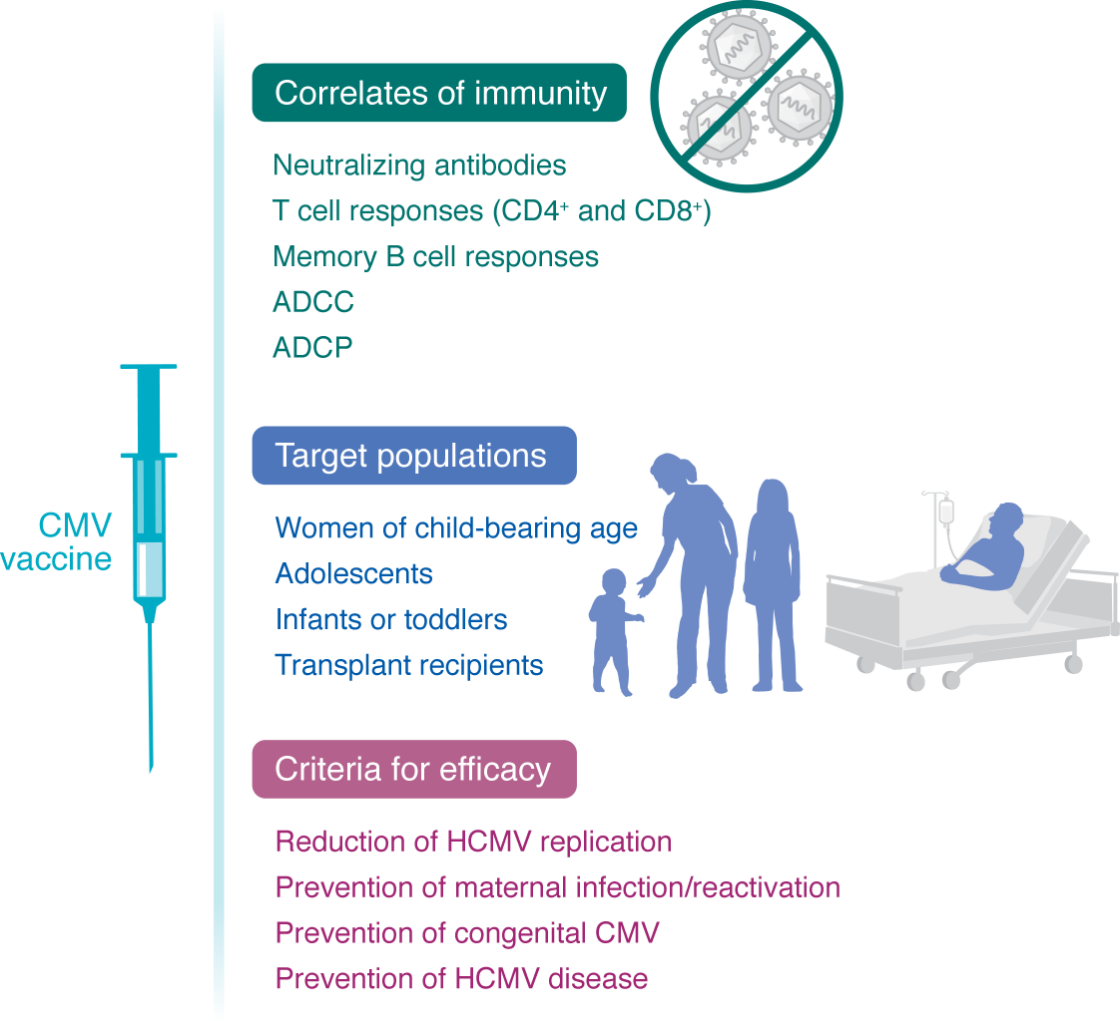
Schematic of the types of immunity, target population, and endpoints for efficacy of a CMV vaccine. Potential immune correlates of protection, the potential target populations, and possible endpoints to establish efficacy for CMV vaccine candidates are listed.

HCMV is a complex organism, and protection against infection requires multiple types of immune response. Demonstration of vaccine efficacy is therefore complex (Figure 1). With this background in mind, the explicit scope of this Review is to summarize the progress in development of an HCMV vaccine, with a particular emphasis on vaccine candidates that are actively in clinical trial evaluation, drawing upon information presented at a conference sponsored recently by the NIAID. The challenges and prospects for developing HCMV vaccines in both the transplant (HSCT, SOT) and cCMV settings are discussed, along with high-priority areas for future research.

## The clinical need for an HCMV vaccine

HCMV infection is the most common congenital infection globally and the most important infectious cause of pediatric disability, including sensorineural hearing loss (SNHL), and yet the virus is ubiquitous in the human population (10, 11), with seropositivity ranging from 60% of adults in industrialized countries to close to 100% of adults in low- and middle-income countries (12, 13). In healthy individuals, an initial HCMV infection may cause a mononucleosis-like syndrome (14, 15), but primary infections are very often asymptomatic/subclinical or may present simply as an undifferentiated febrile ailment (14). Rates of acquisition of infection are increased in breast-fed infants (16); in toddlers (and parents of toddlers) attending group day care (17–21); and in sexually active adolescents and young adults (22–24). It is rare for breast milk–acquired infections to cause disease, with the exception of that in premature infants, in whom acquisition of HCMV from milk can produce a “sepsis syndrome” (25), characterized by thrombocytopenia, neutropenia, and hepatitis. Although the link to adverse neurodevelopmental sequelae for these postnatally acquired infections in premature infants is unproven, there are studies suggesting that such infections may confer an increased risk of SNHL (26).

Like other herpesviruses, HCMV can enter a period of latency after the initial infection and reactivate episodically over the life course (27, 28). During reactivation, viral shedding at mucosal services is common but virtually always asymptomatic. Important sites of latency include the progenitor cells of the myeloid leukocytic lineage, such as CD34^+^ cells and their CD14^+^ derivatives (29). HCMV reactivation and attendant disease are particularly common when the host immune system is suppressed, such as in HSCT recipients (30, 31), SOT recipients (32), and in individuals with other (often iatrogenic) sources of immunosuppression (28, 33). These distinct clinical scenarios, and the implications of these manifestations of infection for vaccine development, are considered in more detail below.

## Congenital HCMV transmission

Global studies have consistently highlighted the potentially debilitating consequences of intrauterine HCMV transmission, also referred to as vertical transmission. cCMV, which occurs in up to 1 in every 200 live born infants, is a pervasive global cause of childhood hearing loss and neurodevelopmental disability as well as other conditions such as hepatitis and seizure disorder. Up to a quarter of congenitally infected infants will have a life-long disability due to fetal infection (34, 35). In the US alone, it is estimated that 20,000 such infections occur annually, with up to 5,000 of these infections resulting in disabilities (36), costing the US health system up to $4 billion annually (37, 38). cCMV is the most common infectious cause of SNHL, estimated to be the cause of 25% of all childhood hearing loss by age 4 years (39). Furthermore, cCMV infection has recently been associated with an increased risk of acute lymphoblastic leukemia (40–43), potentially making childhood cancer prevention another reason for the urgent need for an HCMV vaccine.

As noted above in the Introduction, preconception maternal immunity partially protects infants from vertical HCMV transmission, as demonstrated by a higher rate of congenital transmission and fetal injury after viral infection in seronegative (i.e., newly infected) women compared with seropositive (i.e., infected prior to pregnancy) women (44). However, preconception seropositivity does not confer complete protection against reinfection and subsequent transplacental transmission, and most cCMV infections occur in the setting of preexisting immunity (45). Since the prevalence of cCMV is directly, and not inversely, proportional to the seroprevalence of HCMV in the reproductive population, there is a disproportionate impact of cCMV in children from low-income and underrepresented populations (46–48). This “apparent paradox” (49) of maternal serostatus as a risk factor for cCMV suggests that ultimately the control of cCMV will require immunization of seropositive women, toward the goal of boosting preexisting immunity. Previous consensus panels have identified prevention of maternal infection as the yardstick for licensure of a cCMV vaccine (50), but little attention has been given to the issue of vaccinating seropositives. Arguably, a cCMV vaccine could have its most substantial public health impact in low- and middle-income countries, where the seroprevalence of CMV and therefore the prevalence of cCMV is at its highest. These issues are considered in greater detail later in this Review.

## HCMV-related complications in transplantation

For the SOT and HSCT settings, a variety of benefits could be achieved through licensure of a vaccine for HCMV. HCMV replication following SOT and HSCT has a broad impact on transplant outcomes. Infection contributes to end-organ disease (viremia, pneumonitis, enteritis, and retinitis) and has secondary effects such as increased risk of opportunistic fungal infections, graft-versus-host disease, and graft failure (51). HCMV-related complications have been estimated to increase health care costs by up to 49% for SOT recipients (52). In contrast to maternal-fetal HCMV transmission, where nucleoside antivirals are relatively untested (53, 54), high-risk populations have benefited from the advent of antivirals, with profound effects on HCMV-related morbidity and mortality. Ganciclovir and valganciclovir are currently the drugs of choice for the prevention and treatment of HCMV in HSCT and SOT recipients (32, 55–58). However, the use of antivirals is limited by significant toxicities, including myelosuppression, predominantly neutropenia, and acute kidney injury as well as the development of drug-resistant viral strains (51). This provides a powerful rationale for the development of a HCMV vaccine strategy for SOT and HSCT recipients.

## Other benefits of HCMV vaccination

In addition to the potential association between cCMV and pediatric leukemia (40–43), acquisition of HCMV infection over the life course has been putatively linked to several adult-onset cancers, including glioblastoma multiforme, breast cancer, colon cancer, and prostate cancers (59, 60). HCMV-seropositive status is associated with an enhanced risk of all-cause mortality (61, 62) and with cardiovascular diseases, including atherosclerosis (63). There is a past history of HCMV infection as a cofactor in the pathogenesis of disease due to *Mycobacteria tuberculosis* (64–66) and also as a cofactor in the progression of HIV-associated opportunistic infection, including meningitis caused by *Cryptococcus neoformans* (67). Latent CMV infection exerts a major influence on the immune system during aging, potentially contributing to immunosenescence and the pathogenesis of age-related diseases (68). There are some studies suggesting that HCMV seropositivity reduces the immune response to influenza (69) and COVID-19 (70) vaccination. Patients with critical illnesses requiring intensive care unit (ICU) admission have an increased likelihood of adverse outcomes, including death, if they are HCMV seropositive, which correlates with the magnitude of DNAemia that occurs during their ICU hospitalization (71, 72). For all of these reasons, routine universal immunization against HCMV may confer a broad benefit on human health.

## HCMV biology and host immune response

HCMV is a member of the human *Herpesviridae*, a family of 8 related viruses (73) that share basic genetic (double-stranded DNA genomes), morphologic (enveloped virus particles), and epidemiologic (human-to-human transmission) features. Of the human *Herpesviridae*, HCMV has the largest and most complex viral genome, encoding, as noted above, many homologs of human immune modulation genes (74). The HCMV genome undergoes extensive intra- and intergenic recombination, contributing to a high mutational frequency akin to that of an RNA virus (75–77). This resulting extensive level of strain diversity contributes to the phenomena of reinfection and complicates vaccine development.

Immunity against HCMV is complex, including neutralizing and nonneutralizing effector antibody responses as well as NK and T cell functions. It is unknown why reinfections occur despite preexisting immunity; HCMV strain variation, the quantity of viral inoculum upon exposure, and the capacity of the virus for cell-cell spread may all play roles in enabling reinfection. HCMV vaccine development is complicated by the fact that immunity conferred by natural infection is, at best, imperfect. HCMV encodes a plethora of immune modulation genes that interfere with the host response to infection. These include gene products that downregulate MHC class I molecules on the cell surface, interfering with the CD8^+^ T cell response (78); proteins (79–81) and a microRNA (82) that modify the NK cell response; homologs of human cytokine genes, including IL-10 (83–85); chemokines and G protein–coupled chemokine receptors that modulate the inflammatory response (86, 87); and FCγ IgG receptors that interfere with antiviral host immunoglobulin functions (88). As noted above, HCMV establishes latency (28) and reactivates frequently over the life course, particularly in the context of intercurrent illness or immune suppression (71). These factors contribute to the phenomena of reinfection, which can occur in pregnant individuals (45) or in immunosuppressed HSCT and SOT recipients (89, 90). In the context of pregnancy, congenital HCMV transmission can occur even in the context of preconception immunity. Indeed, the prevalence of cCMV transmission is highest in high-seroprevalence populations, and such infections can lead to disabilities (91). In pregnant women, the relative contributions of reinfection with a new strain of HCMV (92) versus reactivation of a latent infection (93, 94) indicate that the risks of cCMV transmission have not been fully elucidated.

## Antibody responses

Subunit, DNA, mRNA, and viral-vectored vaccines (discussed below) have mainly focused on envelope glycoprotein complexes that are targets for neutralizing antibodies (44, 95). These include glycoprotein B (gB), the “fusion glycoprotein” that mediates viral entry into cells by fusing the virion envelope with the cell membrane, and two additional complexes, the trimeric complex (TC), which comprises glycoproteins H, L, and O (gH, gL, and gO), and the pentameric complex (PC), which comprises gH/gL complexed with UL128, UL130, and UL131A. The TC and PC interact with cell surface receptors and then communicate signals to gB to initiate membrane fusion (96). The relative importance of these complexes in viral tropism and cell entry is unresolved, but both have become cornerstones of subunit vaccine design. In vitro evidence suggests that gB is required for HCMV infection of all cell types, and neutralizing antibodies against gB are presumed to contribute to prevention of infection of most if not all cell types in vivo. In contrast, the PC is dispensable for the infection of fibroblasts but required for entry into epithelial, endothelial, and myeloid lineage cells (97–103). Thus, PC-specific antibodies may be advantageous owing to their extraordinary potency (up to 1,000-fold higher than antibodies targeting gB or TC), but this neutralizing activity is limited to epithelial, endothelial, and myeloid cells, with no activity preventing entry into fibroblasts (104–106). Yet, a recent report indicated that the PC is not required for placental CMV transmission in a nonhuman primate cCMV model (107). Subunit vaccines have focused largely on gB, alone or in combination with the TC and/or PC (108–111). However, TC is not required for HCMV entry into mucosal epithelial cells, and therefore, TC-specific antibodies may block HCMV at mucosal surfaces.

## T cell responses

T cell responses are essential for protection against HCMV-associated disease, as observed in people with AIDS and organ transplant recipients, yet there is less clarity around their role in protection against virus acquisition. Most HCMV-specific CD8^+^ T cells are terminally differentiated and exhibit immediate effector function with a short life span; mucosal tissue, which represents a site of HCMV reactivation, is primarily populated by tissue resident–memory T cells, which do not recirculate (112). The pp65 (ppUL83) tegument protein is the immunodominant CD8^+^ antigen (113). CD8^+^ T cell responses to HCMV often also include IE-1 as well as structural, early/late antigens, and virus-encoded immunomodulatory proteins such as pp28, pp50, gH, gB, US2, US3, US6, and UL18 (114). In total, more than 151 HCMV ORFs were immunogenic for CD4^+^ and/or CD8^+^ T cell responses (115). CD4^+^ T cell responses have also been identified as critical for protection against disease in SOT recipients as well as against congenital infection in humans and in animal models (116, 117).

With respect to the specific role of T cells for cCMV infection, relatively little is known about their role in protection against maternal-fetal CMV transmission (118–120). Recently, an ex vivo model of HCMV infection utilized samples of decidua collected from women with and without preconception immunity to CMV (121). Explanted decidua from women with preconception immunity to HCMV exhibited intrinsic resistance to HCMV and rapidly mounted CD8^+^ and CD4^+^ T cell responses upon HCMV reinfection. The role of CD8^+^ T cells in protecting the maternal-fetal interface from nonprimary HCMV infections is unclear, as HCMV-specific decidual CD8^+^ T cells express a distinct profile of cytolytic molecules compared with that of circulating T cells (122). Other studies have demonstrated lower cytokine and CD4^+^ T cell responses in maternal-fetal dyads where cCMV transmission occurs (123–125). A GPCMV T cell vaccine targeting GP83 (the GPCMV homolog of HCMV ppUL83) was found to be partially protective against cCMV transmission (126). Thus, vaccine-induced T cell responses require further evaluation as a protective component of preconception immunization.

## Nonneutralizing antibody responses 

## and NK responses

NK cell functions are also increasingly recognized as important in HCMV immunity, and infection leads to memory NK cell populations (127, 128) as well as induction of NK-like T cells (129, 130), but their role in vaccine-elicited protection remains undefined. The importance of NK responses is suggested by recent studies that suggest that protection against primary maternal HCMV infection is in part mediated by so-called “nonneutralizing” or “Fc-mediated” immune mechanisms, such as antibody-dependent cellular phagocytosis (ADCP) or antibody-dependent cellular cytotoxicity (ADCC) (131–134). Both mechanisms involve antibodies that bind to viral proteins on the surface of infected cells. Engagement of the exposed antibody Fc domains with Fc receptors on effector cells then facilitates either ADCP (if the effector cell is phagocytic) or ADCC (if the effector cell is an NK cell), in both cases resulting in destruction of the infected cell. HCMV proteins UL16 and UL141 are targeted by ADCC (135). ADCC in HCMV tends to favor late gene products (136), an important issue for the first-generation V160 replication-defective vaccine, where the late viral antigens may be suboptimally expressed (137). A third Fc-mediated mechanism, that of complement-dependent cytotoxicity, has been poorly studied in the context of cCMV. Complement enhances the neutralizing potency of HCMV-seropositive human sera by up to 4-fold and for some gB-based vaccines by up to 100-fold (138). Given that several gB monoclonals neutralize only in the presence of complement (139), such vaccines may exhibit a bias toward complement-dependent epitopes.

## Immunity and reactivation from latency

In addition to preventing primary infection, it is also of interest to consider how an HCMV vaccine might target latent infection, toward the goal of preventing reactivation. For other *Herpesviridae* (including Varicella-zoster virus [VZV] and herpes simplex virus [HSV]), immunization strategies specifically targeting reactivation from latency are described (140). Prevention of reactivation from latency is of course the hallmark of vaccination against herpes zoster (shingles). The adjuvanted VZV gE subunit vaccine (HZ/su) confers higher protection against herpes zoster than the live attenuated vaccine (140), likely attributable to an enhanced memory T cell response (141). For HSV, similar strategies that enhance tissue resident–memory T cell responses appear valuable in preventing reactivation from latency infection in animal models (142, 143) but have not yet been assessed in human clinical trials. A similarly successful HCMV vaccine capable of preventing reactivation from latency could be useful at limiting reinfections in high seroprevalence populations. The targets of such an approach are only speculative at this time, but the characterization of several HCMV-encoded ORFs, for example, *UL133-138*, that play key roles in both establishment of and reactivation from latency (144) could, in principle, become the basis for subunit vaccine design. Notably, a replication-impaired, disabled, infectious single-cycle (DISC) virus HCMV vaccine currently in clinical trials, V160 (discussed later in this Review), lacks these ORFs (145). Studies of HCMV latency, in contrast to VZV and HSV, demonstrate that a wide range of viral late gene transcripts are actively expressed, albeit to lower levels than during primary infection (146). Future studies focused on enhancing the broadest and most sustained T cell response to the repertoire of HCMV antigens expressed throughout the viral life cycle could be of value, as well as the investigation of strategies that enhance innate immunity (147), in generating a vaccine capable of reducing reactivation of latent infection.

## Summary — required attributes for an HCMV vaccine

As natural immunity is not always protective against HCMV reinfection or congenital transmission, the goal of a successful HCMV vaccine will be to elicit immunity that improves on that elicited by natural infection. Current vaccine trials will evaluate the possibility of protection against a range of HCMV strains. Though there is evidence for antigenic variation, it is not certain whether HCMV vaccines with diverse antigens from multiple strains will be required. For example, immunogenicity data from a multistrain gB vaccine demonstrated limited effect on immunity (148). Another possible result of these vaccine trials will be further definition of the immunologic correlates of protection against placental transmission or disease (149). In particular, the role of mucosal immunity in preventing acquisition of HCMV requires more studies. However, it is our view that current knowledge gaps in vaccine design should not temper the urgency of achieving vaccine licensure to address the unmet need for protecting newborns against congenital HCMV transmission. Indeed, in view of the huge scope of the problem, the licensure of even a moderately effective vaccine would be a major public health success.

## Lessons from recent HCMV vaccine clinical trials

Generally speaking, HCMV vaccines that have advanced to clinical trial testing can be categorized as subunit vaccines (expressing gene product[s] of importance in protective immunity), using an appropriate source of immunogen (protein, DNA, or RNA) expressed de novo or in the context of an expression vector, or whole-virus vaccines (live, attenuated viruses or DISC viruses). In terms of the immunogen(s) of choice, most subunit vaccine candidates have focused on recombinant gB, the major viral fusogen (150–152) encoded by the UL55 ORF, and the ppUL83, which, as noted above, is the dominant T cell immunogen in the context of infection (113). Emphasis has also been placed on the IE1 and IE2 (encoded by UL123 and UL122, respectively) proteins (153).

Various expression platforms expressing combinations of these immunogens have been studied in humans. A DNA vaccine developed by Astellas, ASP0113, contained two plasmids encoding gB and ppUL83. However, this vaccine had no effect on HCMV disease in a phase II study in setting of SOT (154) or in a phase III study in HSCT (155). This vaccine is not being further developed. Another vaccine candidate, HB-101, is based on expression of gB and ppUL83 using a lymphocytic choriomeningitis virus platform. In a placebo-controlled phase I study, 54 participants that received vaccine demonstrated pp65-specific CD8^+^ T cell responses and neutralizing antibody responses; however, the manufacturer has suspended the product development plan for this vaccine (156). A two-component alphavirus replicon particle vaccine, expressing HCMV gB or a pp65/IE1 fusion protein, was evaluated in a randomized, double-blind phase I clinical trial in HCMV-seronegative individuals (157). Antibody and T cell responses were noted, but the vaccine was not further developed.

With respect to whole-virus vaccines, HCMV strains were engineered in a fashion that was predicted to increase the immunogenicity of the Towne strain by generating genomic reassortments of Towne sequences mixed with a minimally passaged clinical isolate of HCMV, the “Toledo” strain. Four recombinants were tested in phase I studies of small numbers of participants, and one turned out to be suitably immunogenic (158). These so-called “Towne-Toledo chimeras” did not undergo any further testing; one potential deficiency in the platform is that none of the chimeras encode a functional PC (159). None of these chimeric vaccines demonstrated any evidence of “boosting” anti-HCMV immune responses in a phase I study in HCMV-seropositive recipients (160).

## HCMV vaccines currently in clinical trials

Focusing on platforms that have advanced beyond phase I study, several vaccines have demonstrated varying degrees of protection against HCMV infection and/or disease in efficacy studies (Table 1). Early clinical trials of a live, attenuated HCMV vaccine in kidney transplant recipients demonstrated good protection against disease, but little effect on viral acquisition (161). Subsequently, an adjuvanted protein subunit vaccine based on the primary viral fusogen, gB, conferred partial protection against virus acquisition in seronegative adolescents and postpartum women in two distinct phase II trials (108, 109), one as a single center in postpartum women and the other as a multicenter trial in adolescent girls, which respectively demonstrated 50% and 43% efficacy of the gB vaccine. Furthermore, the gB vaccine reduced HCMV viremia and the need for antiviral therapy in HCMV-seronegative kidney and liver SOT recipients from HCMV-seropositive donors (95). While the gB vaccine was not licensed because its prevention of infection was modest and induction of neutralizing antibodies was limited, the efficacy result did not fully take into account the volume of the inocula of a transplanted organ, viremia levels, or related disease. Thus, a primary goal of HCMV vaccine development is to improve on the prior achieved efficacy and/or demonstrate higher efficacy using a virologic or disease outcome endpoint.

Other subunit vaccines based on envelope glycoprotein complexes are in clinical trials. The safety and immunogenicity of an enveloped virus-like particle vaccine expressing gB, with or without alum adjuvant, was evaluated in a placebo-controlled study in 125 participants (NCT02826798). The highest antibody titers were in the alum-adjuvanted 2.0 μg dose group (162). Several studies of an mRNA-based HCMV vaccine encoding gB and PC, mRNA-1647 (111, 163, 164), are in progress (Table 1), including a phase III efficacy study (NCT05085366), with results anticipated in 2025. An interesting expression platform using Hepatitis B SPYTag/SPYCatcher, and expressing the HCMV PC (NCT06145178), has been developed (Table 1). The manufacturer, SpyBiotech, recently completed participant enrollment in a phase I HCMV study of this vaccine, SPYVLP01, in 120 healthy adults, aged 18–50 years, over a six-month dosing schedule. Results have not yet been reported. Using the MVA vaccinia virus as vector carrying the pp65 protein plus IE proteins 1 and 2, a vaccine called Triplex showed promise against HCMV infection and reactivation in the allogeneic transplant setting in a published phase II trial (165). As a result, several new clinical trials have been ongoing or completed (see NCT06059391, NCT03354728, NCT05099965) that expand on the original design concept (166, 167). A multicenter phase II study of Triplex vaccine for “Control of CMV in Patients Undergoing Liver Transplantation” (COLT trial) is currently ongoing (NCT06075745). The V160 vaccine (described below) could also be considered for the transplant setting (168).

In the live, attenuated vaccine category, recent progress has been made. A whole-virus (but replication-incompetent) vaccine was recently evaluated in a phase II trial in women of child-bearing age. The vaccine (as noted above) is a DISC vaccine, V160 (145, 169, 170). The efficacy against primary HCMV infection in the phase II trial (NCT03486834) was 42% (168). Although the reported efficacy was suboptimal, case definition in this study was based on any detection of HCMV DNA in mucosal fluids in postvaccination follow-up, rather than the presence of systemic infection. Transient detection of viral DNA at mucosal sites may overestimate true acquisition events, resulting in an underestimation of vaccine efficacy (168). Furthermore, perhaps the endpoint of efficacy trials should be prevention of HCMV transmission to the fetus, not solely the prevention of maternal infection. It is currently unclear if clinical development of the DISC V160 vaccine candidate will continue.

## Gaps in knowledge: challenges for HCMV vaccine design

An important limitation hindering HCMV vaccine development is the gap in our understanding of what components of the immune response would protect against mucosal virus acquisition versus systemic HCMV disease and transfer to the fetus. Immune correlate analyses of the partially protective gB subunit vaccine trials revealed that plasma IgG recognition of gB expressed on the surface of a cell, but not recognition of the soluble gB protein, predicted protection against HCMV acquisition (131). Thus, it is likely that native conformation of gB, as well as other glycoproteins, is critical to elicitation of protective immune responses. While the partially protective DISC V160 vaccine presented membrane-associated glycoprotein antigens, including gB, the magnitude of the immune responses elicited by this alum-adjuvanted vaccine may have limited its efficacy. Vaccines that combine glycoprotein antigens beyond gB, such as the PC that is critical to viral entry into endothelial and epithelial cells, have been assessed in animal models (171, 172) and are currently the basis of the Moderna mRNA-1647 vaccine that is in phase III trials (164, 173). While the high levels of neutralizing antibodies generated by the inclusion of the PC did not generate highly protective responses against mucosal virus acquisition (174) and were not required for prevention of placental infection in nonhuman primate models (107), it is possible that this approach will show more benefit when studied in humans, which may involve lower levels of virus in the natural setting, as opposed to the challenge doses and routes of inoculation used in animal modeling.

The recent success of structurally engineered viral fusion proteins in the development of respiratory syncytial virus and SARS-CoV-2 vaccines reveals the importance of applying structure-based design to HCMV gB and its entry complex partners. Immunization with a fully trimeric (175) or “prefusion” gB conformation (151) has the potential to enhance entry-blocking activity of vaccine-elicited antibodies, though current immunogenicity results in mice have only shown that prefusion gB vaccines elicited neutralizing antibodies equivalent to those conferred by postfusion gB vaccines (176). Moreover, although the field has focused on neutralizing functions, nonneutralizing effector antibody functions such as ADCC and ADCP may be more important than virus-neutralizing antibody responses in mediating protection (177). As there is evidence that T cell responses are likely also essential for both prevention of congenital infection and reduction of complications in SOT and HSCT recipients (123, 178), current vaccine approaches also should focus on the elicitation of T cell immunity. As discussed above, HCMV also employs a number of immune evasion mechanisms to escape host immune responses, such as NK receptor ligand intercepting molecules (179), viral cytokines (180), and viral Fc receptors (181, 182), and novel vaccine strategies are needed to counteract the viral immune evasion functions that contribute to reinfections. Thus, there is a potential to include immune evasion antigens in HCMV vaccine design to elicit blocking antibody responses that can enhance the effectiveness of vaccine-elicited anti-HCMV immunity over that of natural immunity.

## Next steps for developing a cCMV vaccine

The key questions for a vaccine to prevent congenital infection are as follows: (a) how can next-generation HCMV vaccine candidates improve on the nearly 50% efficacy against acquisition of maternal infection in studies reported to date; and (b) what will be required to demonstrate protection against fetal infection and/or sequelae, even if protection against maternal infection is incomplete? As discussed above, maximizing the neutralizing antibody response, as well as the nonneutralizing responses, such as ADCP, in variety of cell types may be achievable by targeting the combination of gB and PC. Additionally, individual pentamer subunits may elicit cellular immune responses that could augment the protection conferred by antibody responses (173). The importance of antipentamer responses in protection against horizontal and congenital transmission has not been fully demonstrated, but an ongoing phase III trial of the mRNA-1647 vaccine encoding both gB and the PC will provide insight into the success of this combined strategy, as it pertains to horizontal transmission (164).

The target populations for HCMV vaccines intended to prevent cCMV infection need to be defined. The National Academy of Medicine modeled a vaccine for cCMV against a target population of 12-year-old boys and girls (183). Candidates for immunization potentially include seronegative individuals before pregnancy, adolescents, and infants or toddlers (183, 184). Toddlers are a particularly important population to consider, since enrollment in group daycare — where CMV transmission frequently occurs — stands as a major risk factor for their parents to acquire the infection via exposure to infectious secretions and body fluids. Inasmuch as the majority of worldwide congenital HCMV infections occur in the fetuses of seropositive individuals who could undergo vaccination to prevent HCMV reinfection or reactivation, a vaccine that enhances preconception immunity is of key importance. A licensed vaccine against congenital HCMV that is useful both in seronegative and seropositive people would be an enormous advancement for public health.

## Developing a vaccine against HCMV-associated complications of transplantation

In addition to causing congenital infection, HCMV is one of the most frequent serious infections of patients receiving SOT or HSCT as a result of the immunosuppression that is necessary in these settings. Although the correlates of protection may be different than those that prevent infection of a developing fetus, conferring pretransplant HCMV immunity to patients undergoing transplantation could provide substantial benefits that enhance transplant outcomes, reduce mortality, and protect against the indirect effects of HCMV. HCMV disease occurs both in seronegative transplant recipients undergoing primary infection and also in seropositive recipients due to reactivation of the latent virus. Early studies of an attenuated HCMV strain given to recipients before transplantation showed significant elimination or reduction of disease in both groups (185, 186). However, concerns about the safety of a live vaccine virus shifted studies of HCMV disease prevention to the use of subunit protein antigens (187) and to those same antigens presented by poxvirus vectors (188, 189). Partial success in reduction of HCMV disease after transplantation was accomplished with both approaches. Yet, a gap in the field has prevented the continued development of partially effective vaccines: how do we define the ultimate goal of a HCMV vaccine? Sterile protection may be difficult, but prevention of dissemination in the body and to the fetus may not be.

The CMV vaccine antigen most studied in the setting of SOT is the surface gB glycoprotein, which by itself has shown efficacy in preventing acquisition of HCMV in both seronegative women and also in seronegative transplant recipients (109, 187, 190). Thus, it should be part of any successful HCMV vaccines used in the setting of SOT. However, a T cell–based vaccine is likely necessary to better protect transplant recipients. A vaccine containing gB protein in combination with several CD8^+^ T cell epitopes (Table 1) has demonstrated good B and T cell responses in animals (191). Inclusion of ppUL83 may be desirable for immunization of both HCMV-seronegative and -seropositive patients (192).

As noted above, mRNA technology has recently been applied to the development of HCMV vaccines. The gB protein presented by mRNA induced stronger ADCC and a greater breadth of gB-specific T cell responses than the adjuvanted protein (164, 193). Thus, we now have multiple ways of presenting HCMV antigens to the immune system, which allows induction or enhancement of antibody and cellular immune responses in both seronegative and seropositive individuals (194). Ongoing and completed trials in the SOT and HSCT setting have focused on therapeutic vaccination to prevent or reduce viremia. Evaluation of these vaccine candidates against HCMV-related disease in transplant recipients is an important next step in the development pipeline, drawing on comparisons to studies performed in parallel with vaccines primarily focused on prevention of cCMV (Table 1).

## Summary

In summary, the 2023 NIAID meeting proceeding revealed that the recent entry into clinical trials of multiple HCMV vaccines gives hope that intrauterine infection and transplant complications can be substantially reduced in the near future (195). It was noted that investigations of immune correlates in human populations and mechanistic studies of HCMV disease in preclinical models should continue and should be related to the responses elicited by distinct HCMV vaccine platforms. Further emphasis was placed on defining virologic and disease outcome measures for ongoing vaccine assessments. A consensus conclusion of this meeting of HCMV vaccine experts was that if current vaccine candidates in late-stage trials result in the safe protection of the majority of vaccinees against HCMV infection and/or disease, they should be reviewed for licensure. Even partial success of these candidate vaccines against HCMV is likely to have an enormous effect in reducing CMV-associated congenital disease in children as well as disease in SOT and HSCT recipients globally, and acceleration of promising candidates through the regulatory pathway should be a high priority.

## Figures and Tables

**Table 1:**
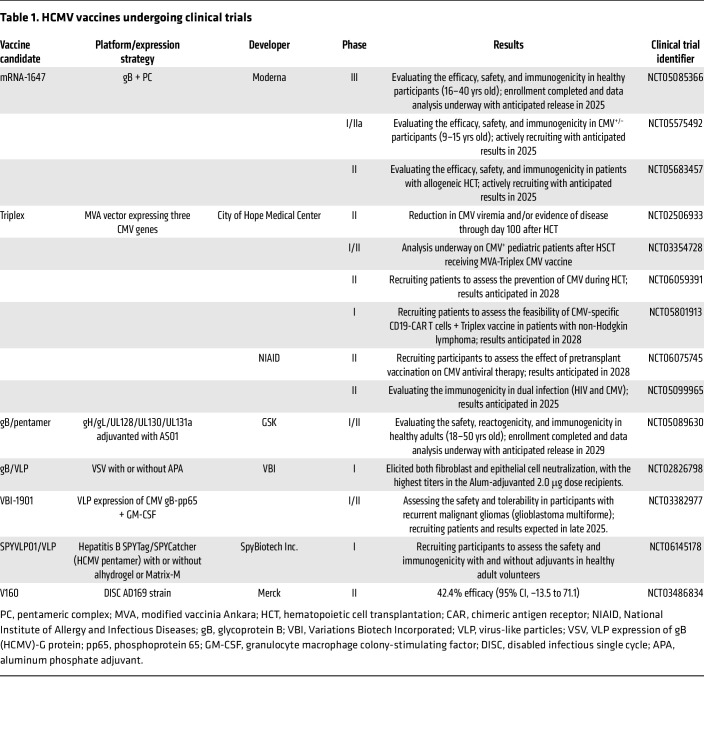
HCMV vaccines undergoing clinical trials

## Version 1 01/02/2025

Electronic publication
